# **Glycated albumin as a diagnostic tool for diabetes mellitus in transfusion-dependent β-thalassemia patients**

**DOI:** 10.1007/s12020-025-04410-9

**Published:** 2025-09-09

**Authors:** Gianluca Marzi, Martina Verrienti, Filomena Longo, Camilla Alice Cattaneo, Alberto Gobbo, Katalin Vetralla, Gian Pietro Franzè, Alberto Cossu, Martina Culcasi, Maria Chiara Zatelli, Maria Rosaria Ambrosio

**Affiliations:** 1 Section of Endocrinology, Geriatrics and Internal Medicine, Department of Medical Sciences, University of Ferrara, Ferrara, Italy; 2 Unit of Endocrinology and Metabolic Diseases, Department of Specialty Medicine, Azienda Ospedaliero Universitaria S.Anna, Ferrara, Italy; 3 Day Hospital of Thalassemia and Hemoglobinopathies, Department of Specialty Medicine, Azienda Ospedaliero Universitaria S. Anna, Ferrara, Italy; 4 Integrated Activity Department, Imaging and Laboratory Diagnostics, Clinical Pathology, Azienda Ospedaliero Universitaria S. Anna, Ferrara, Italy; 5 Radiology Unit, Azienda Ospedaliero-Universitaria di Ferrara, Ferrara, Italy

**Keywords:** Beta thalassemia, Transfusion, Iron overload, Diabetes, Glycated albumin

## Abstract

**Introduction:**

β-thalassemia is an inherited hemoglobinopathy which, in severe cases, requires lifelong transfusions, leading to iron overload and increases risk of metabolic complications, including diabetes mellitus. The standard glycemic marker, glycated hemoglobin, is unreliable in transfusion-dependent patients due to altered erythrocyte turnover. This study investigates the possible utility of glycated albumin as an alternative biomarker for diagnosing glucose metabolism disorders in transfusion-dependent patients.

**Methods:**

A cross-sectional analysis was conducted on 254 transfusion-dependent patients followed at the Center for Hemoglobinopathies and Thalassemia at Ferrara Hospital, evaluating Glycated Albumin levels, fasting plasma glucose, oral glucose tolerance test results, and iron overload assessed by Magnetic Resonance Imaging.

**Results:**

Glycated Albumin levels significantly correlated with Fasting Plasma Glucose (*r* = 0.710, *p* < 0.01), Oral Glucose Tolerance Test glucose levels at 60 and 120 min, and the area under the glycemic curve (*p* < 0.01). ROC curve analysis identified a Glycated Albumin cutoff of 14.95% for Diabetes Mellitus diagnosis, with a sensitivity of 93.1% and specificity of 89.1%. Glycated Albumin correlated with hepatic and heart iron accumulation but not with pancreatic iron overload.

**Conclusions:**

These findings indicate Glycated Albumin as a promising marker for glycemic assessment in transfusion-dependent patients, warranting further validation in multicenter studies.

**Supplementary Information:**

The online version contains supplementary material available at 10.1007/s12020-025-04410-9.

## Introduction

**Fig. 1:**
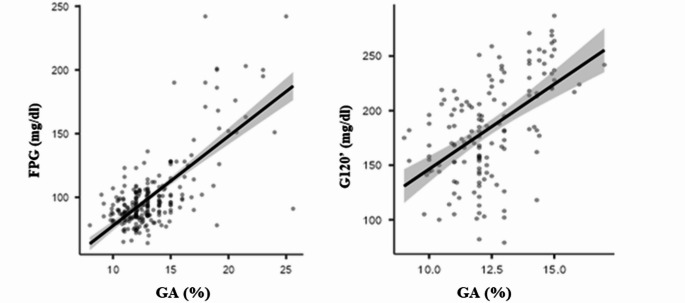
GA correlation with FPG (**A**; r = 0.710, p < 0.01) and glucose levels 120 min (G120’) after glucose load (**B**; *r* = 0.612, *p* < 0.01)

β-Thalassemia (βT) is an inherited blood disorder characterized by reduced production of β-haemoglobin chains [1]. Transfusion-dependent β-T (TDT) patients rely on lifelong blood transfusions, leading to iron overload that accumulates in several organs (liver, heart, and pancreas) [2, 3]. As a consequence, endocrine and metabolic complications such as diabetes mellitus (DM) may develop [4]. Consistent and regular chelation treatment can help reduce complications risk, while poor adherence may compromise its effectiveness and contribute to endocrine dysfunction progression [5, 6]. The pathophysiology of glucose metabolism disorders in TDT patients is multifactorial and remains incompletely understood (Fig. 1, Supplementary Materials). However, iron accumulation in β cells is thought to induce oxidative stress, promoting β cell apoptosis through ferroptosis, leading to reduced insulin secretion [7]. Simultaneously, iron deposition in peripheral tissues, particularly liver and muscle, disrupts glucose homeostasis [8]. Hepatic iron overload, often exacerbated by viral co-infections such as hepatitis B and C, impairs insulin clearance and promotes insulin resistance [9]. The dual impairment of insulin production and sensitivity distinguishes DM in thalassemia from the traditional type 1 or type 2 DM, making its management more complex. DM prevalence in βT population generally ranges between 5 and 36% [10], depending on the studied population and iron overload management protocols. Unlike the general population, a higher incidence of glucose metabolism disorders is observed as early as the first two decades of life [11]. Typically, glycated hemoglobin (HbA1c) is used to assess long-term glycaemic control, reflecting glucose levels over the previous 2–3 months. However, HbA1c reliability is compromised in thalassemia patients due to altered red blood cell lifespan and frequent transfusions [12, 13].

Glycated albumin reflects glycation at serum albumin amino-terminal end, primarily on lysine and arginine residues [14]. This product can be measured chromatographically or enzymatically, providing an estimate of glucose levels over ~ 2–3 weeks, corresponding to albumin half-life [15]. The glycated albumin-to-total albumin ratio (GA) offers a reliable assessment of glucose levels, with lower variability than HbA1c, especially in peculiar conditions such as TDT [16, 17]. Previous studies indicate that GA is less influenced by hematologic abnormalities, supporting the hypothesis that GA may represent an accurate glycaemic marker in TDT population [13]. GA demonstrates promising applications in conditions where HbA1c may be unreliable, such as gestational DM, chronic kidney disease, and hemolytic anaemias [18]. Unlike HbA1c, GA may provide an immediate estimate of median glucose levels, representing a putative valuable tool for monitoring recent changes in glucose levels and treatment response. Most studies on GA have been conducted in Japanese population, where specific cut-off values have been proposed for general population [19]. However, data on βT remain limited, emphasizing the need for further investigations to establish standardized cut-off values and integrate GA into routine clinical practice for this population. Our cross-sectional study explores the potential usefulness of GA in diagnosing DM in TDT patients, aiming to provide a novel diagnostic tool in βT.

## Methods

Our cross-sectional study evaluates TDT patients referring to the Center for Hemoglobinopathies and Thalassemia at Ferrara Hospital, recruited between January 2023 and June 2024. All patients had been receiving regular blood transfusions, maintaining pre-transfusion haemoglobin (Hb) levels between 9.5 and 10.5 g/l. The main inclusion criteria were: diagnosis of β-T, requiring periodic transfusions and age > 18 years. Exclusion criteria were the presence of chronic conditions such as cardiopathy, liver disease or nephropathy that could affect circulating protein levels. Nephropathy was defined as estimated glomerular filtration rate (eGFR) < 60 ml/min per 1.73 m² or the presence of macroalbuminuria. Liver function markers considered as exclusion criteria were: albumin levels < 3 g/dl, total bilirubin > 2 mg/dl, or INR > 2. Patients classified as stage IV heart failure according to the New York Heart Association (NYHA) were excluded. Furthermore, patients with a diagnosed DM treated with insulin or other hypoglycaemic drugs were excluded from the study.

Anthropometric measurements, including Body Mass Index (BMI), were performed following standardized protocols. The following biochemical data were considered in the analysis: GA (%), fructosamine (µmol/L), FPG (mg/dl), insulin (µU/ml), creatinine (mg/dl), albumin (g/dl), total cholesterol (mg/dl), HDL-cholesterol (mg/dl) and triglycerides (mg/dl), ferritin (ng/ml) all evaluated by standard methods. Blood samples were taken after overnight fast, right before the next scheduled transfusion. Fasting does not influence GA levels. Pre-transfusion hemoglobin level (Hb) was calculated as the average of the Hb measurements taken over the previous year. GA measurement in plasma samples was performed by the automated iLab Taurus analyzer (Instrumentation Laboratory, Milan, Italy) with the enzymatic colorimetric QuantILab Glycated Albumin assay (Instrumentation Laboratory). According to Good Clinical Practice of the Italian Society of Thalassemia and Haemoglobinopathies in TDT population in screening of glucose metabolism disorders oral glucose tolerance test (OGTT) should be performed once a year [20]. OGTT was performed following standard procedures; additional blood samples were collected at 30, 60, 90 and 150 min after glucose load. Area Under the Glycaemic Curve (AUCG) was determined using the trapezoidal rule. In accordance with the latest American Diabetes Associations (ADA) guidelines, a fasting glucose level > 125 mg/dL or a glucose level ≥ 200 mg/dL two hours after glucose load under OGTT were considered indicative of DM [21]. The Homeostasis Model Assessment (HOMA-IR) index [22], which correlates with insulin resistance, and HOMA-β%, which correlates with β-cell function [23], were calculated using FPG and fasting insulin levels. The eGFR was calculated using the Cockcroft-Gault formula.

All MRI exams were performed on a 1.5-T scanner (Magnetom Aera, Siemens Healthineers, Germany). A cardiac phased-array receiver surface coil with breath-holding in end-expiration and ECG-triggering was used. Myocardial iron overload (MIO) was assessed by acquiring three parallel short axis (SAX) slice of the left ventricle (LV) with a T2* gradient–echo multiecho sequence. Each slice was acquired at 10 echo times (TEs 2.0–22.34 ms) with an echo spacing of 2.26 ms [24].

For liver and pancreas iron overload assessment, a breath-hold T2* gradient-echo multiecho sequence was used [25]. A mid-hepatic slice and multiple axial slices of the upper abdomen, covering all the pancreatic parenchyma, were obtained at 10 TEs (TEs 1.08–21.42 ms) with an echo spacing of 2.26 ms. Image analysis was performed using a custom-written, previously validated software (HIPPO MIOT^®^) [26]. Liver T2* values were quantified in a circular region of interest (ROI) of fixed dimension delineated bypassing the blood vessels and converted into liver iron concentration (LIC) using the Wood’s calibration curve [27, 28]. Three small ROIs were manually drawn over the pancreatic head, body and tail avoiding large vessels and susceptibility artifacts produced by colic or gastric intraluminal gas. Global pancreatic T2* value was obtained by averaging the T2* values from the three regions. Cardiac T2* was assessed in the mid interventricular septum and in all the AHA/ACC 16 LV segments [29]. All measurements were performed by experienced radiologists.

### Statistical analysis

Categorical quantitative variables are expressed as absolute values and percentages; continuous variables will be presented as mean ± standard deviation (SD) for normally distributed data, whereas median and interquartile range (IQR) will be used for non-normally distributed variables. Qualitative variables were transformed into dichotomous and/or polytomous categories. Shapiro-Wilk test was performed to assess the normality of the continuous variables. The association between categorical variables was assessed using Chi-square test. Additionally, the Student’s t-test was used to compare two groups for the same continuous quantitative variable. Correlation analysis between two continuous quantitative variables was performed using Pearson’s correlation. Univariate and multivariate linear regression models were employed to evaluate the relationships between the independent variables and AG. Using receiver operating characteristic (ROC) curve analysis and the Youden index, the optimal cut-off value with the greatest sensitivity and specificity was identified for the considered glycaemic parameters. Statistical significance was considered with p-value < 0.05.

Descriptive data were collected using Microsoft Excel, while statistical functions and tests were conducted with the software Jamovi (version 2.6.13) and with R software (version 4.3.1).

## Results

The study includes 270 TDT patients aged between 18 and 82 years (49.10 ± 10.09 years). 16 were excluded because they did not meet the inclusion criteria or due to unavailable data. Of the remaining 254 patients (109 males and 145 females), 142 underwent an OGTT. All patients received blood transfusions on average every 18 days (median 17.5 days; range 7–29), with a mean pre-transfusion Hb level of 10.2 g/dL (median 10 g/dl, range 8.9–12.9); all of them were adequately treated with chelation agents. Mean GA level was 13.19 ± 2.85%. 52 patients (20.47%) presented FPG levels > 125 mg/dl. Table 1 shows patients characteristics according to sex.

**Table 1:** Patients characteristics

	Total *N* = 254	Female *N* = 145	Male *N* = 109	*p*-value
**Age (years)**	49.10 ± 10.09	49.20 ± 10.40	48.97 ± 9.70	NS
**BMI (kg/m** ^**2**^ **)**	23.12 ± 3.36	22.99 ± 3.40	23.28 ± 3.30	NS
**Pre-transfusion Hb (g/dL)**	10.20 ± 0.76	10.20 ± 0.76	10.20 ± 0.77	NS
**Transfusion frequency (days)**	18.00 ± 4.10	18.10 ± 3.36	17.90 ± 4.10	NS
**Ferritin (ng/ml)**	791 ± 627	850 ± 623	797 ± 610	NS
**GA (%)**	13.19 ± 2.85	13.35 ± 3.05	12.95 ± 2.56	NS
**Fructosamine (µmol/L)**	314 ± 49.6	309.4 ± 46.6	320.8 ± 52.6	NS
**FPG (mg/dl)**	99.76 ± 27.64	98.99 ± 26.16	100.77 ± 29.55	NS
**Basal Insulin (µU/ml)**	6.73 ± 4.67	6.75 ± 4.50	6.69 ± 4.91	NS
**Homa- IR**	1.76 ± 1.23	1.76 ± 1.24	1.75 ± 1.26	NS
**HOMA-β (%)**	88.56 ± 92.76	86.97 ± 74.09	93.27 ± 80.48	NS
**Total Cholesterol (mg/dl)**	134.95 ± 32.76	135.74 ± 35.73	134.33 ± 34.49	NS
**HDL-cholesterol (mg/dl)**	42.41 ± 12.76	41.45 ± 13.27	40.25 ± 12.10	NS
**Triglycerides (mg/dl)**	104.1 ± 58.97	106.21 ± 59.25	101.11 ± 58.73	NS
**Albumin (g/dl)**	4.39 ± 0.34	4.12 ± 0.32	4.66 ± 0.38	0.042
**Creatinine (mg/dl)**	0.75 ± 0.21	0.71 ± 0.22	0.79 ± 0.19	< 0.001
**eGFR (ml/min)**	108.29 ± 26.13	110.10 ± 29.20	98.13 ± 23.22	NS

Variables are shown as mean ± standard deviation

*NS* not statistically significant, *GA* glycated albumin, *Hb* haemoglobin, *BMI* body mass index, *FPG* fasting plasma glucose, *EGFR* estimated glomerular filtration rate

**Fig. 2:**
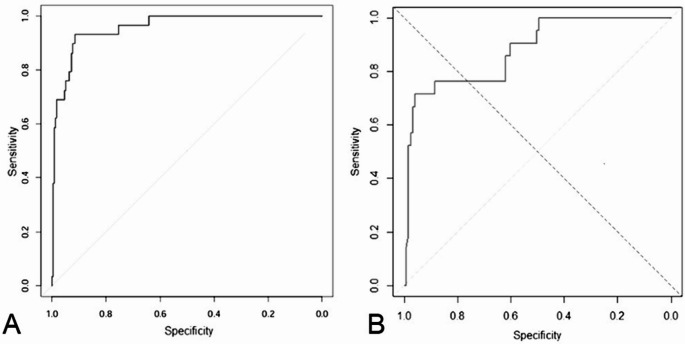
ROC analysis of plasma GA cut-off for detecting newly diagnosed DM according to FPG, adjusted for age **A** and according to OGTT, adjusted for age **B**

No statistically significant differences were observed when comparing GA and FPG levels between males and females. No significant correlation between GA and pre-transfusion Hb levels was found. GA levels were significantly higher in patients with FPG levels > 125 mg/dL as compared to those with FPG ≤ 125 mg/dL (18.46% vs. 12.67%, *p* < 0.01). (Fig. 2A, Supplementary materials). Additionally, individuals with a BMI ≥ 25 kg/m² had higher GA levels compared to those with a BMI < 25 kg/m² (14.19% vs. 13.62%; *p* < 0.01).

Considering OGTT results, 46 patients (32.39%) showed glucose levels > 200 mg/dL at 120 min post-glucose load. No significant differences in glucose levels at 60–120 min were observed between sexes (Table 1, Supplementary materials). GA levels were significantly higher in patients exhibiting glucose levels ≥ 200 mg/dL at 120 min under OGTT as compared to those with glucose levels < 200 mg/dL (14.28% vs. 12.06%, *p* < 0.01). (Fig. 2B, Supplementary materials).

A weak but statistically significant correlation was also observed between FPG and fructosamine levels (*r* = 0.211, *p* < 0.01) (Fig. 3A, Supplementary materials). No statistical difference was found comparing fructosamine levels in patients with FPG > 125 mg/dl and patients with normal FPG. (Fig. 3B, Supplementary materials)

Univariate linear regression analysis showed that GA levels significantly positively correlated with FPG levels, basal insulin levels, HOMA-IR and age (*p* < 0.01), as well as with AUC_G_ and glucose concentrations at 60 and 120 min under OGTT (Fig. 1).

Furthermore our data show that an increase in creatinine levels and a consequent reduction in eGFR is significantly associated with higher GA levels (*p* < 0.01). The other biochemical parameters did not significantly correlate with GA levels (Table 2).

**Table 2:** Univariate linear regression models with GA as dependent variable

	Estimate	Standard Error	CI 95%	*P* value
**Pre-transfusion Hb (g/dL)**	0.007	0.028	-0.024-0.042	NS
**FPG (mg/dl)**	0.072	0.004	0.063–0.081	< 0.001
**OGTT 60’ (mg/dl)**	0.018	0.002	0.013–0.023	< 0.001
**OGTT 120’ (mg/dl)**	0.021	0.002	0.017–0.026	< 0.001
**AUC**_**G**_	0.030	0.003	0.024–0.036	< 0.001
**Age (years)**	0.074	0.017	0.041–0.108	< 0.001
**BMI (kg/m^2)**	0.118	0.052	0.015–0.221	0.025
**Creatinine (mg/dl)**	2.100	0.842	0.438–3.760	0.013
**eGFR (ml/min)**	-0.019	0.005	-0.029-0.009	< 0.001
**HOMA-IR**	0.723	0.101	0.523–0.922	< 0.001
**HOMA-β**	-0.167	0.002	-0.258-0.025	NS
**Basal Insulin (µU /ml)**	0.121	0.038	0.047–0.195	0.001
**Total Cholesterol (mg/dl)**	0.006	0.005	-0.003-0.016	NS
**HDL-cholesterol (mg/dl)**	-0.011	0.014	-0.039-0.016	NS
**Triglycerides (mg/dl)**	0.006	0.003	0.003–0.122	NS
**Ferritin (ng/ml)**	0.017	0.008	-0.127-0.016	NS
Heart T2* (ms)	0.004	0.019	-0.034-0.041	NS
Liver T2* (ms)	0.009	0.022	-0.036-0.054	NS
Pancreatic T2* (ms)	0.006	0.002	-0.030-0.043	NS
**LIC (mg/g/dw)**	0.002	0.028	-0.052-0.057	NS

*NS* not statistically significant, *FPG* fasting plasma glucose, *BMI* body mass index, *OGTT 60’* glucose level after 60’ from oral glucose administration, *OGTT* 120’ glucose level after 120’ from oral glucose administration, *AUC*_*G*_ area under glycaemic curve, *LIC* liver iron concentration

A total of 182 TDT patients underwent MRI to assess iron accumulation in the last year. No significant correlations were identified between GA levels and iron accumulation in the pancreas, liver, or heart. In particular, patients with T2* values indicative of pancreatic iron overload did not exhibit higher GA levels compared to individuals with no pancreatic iron accumulation. Interestingly, patients showing LIC consistent with moderate to severe iron accumulation presented higher GA levels as compared to patients showing LIC consistent with mild iron accumulation (15.62% vs. 13.80% *p* < 0.01). In addition, GA levels were significantly higher in patients with heart iron overload as compared to patients with low cardiac iron accumulation (15.43% vs. 13.50% *p* < 0.01).

The multivariate linear regression model confirmed that GA is significantly associated with age and FPG levels (Table 3). According to ROC analysis, GA levels of 14.95% represent the best cut-off to predict DM on the basis of FPG (AUC = 0.952, sensitivity 93.1%, specificity 89.1%, Youden index 0.823) (Fig. 2A). Similarly, GA levels of 14.35% represent the best cut-off to predict DM on the basis of glucose levels 120 min after glucose load according to ROC analysis (AUC = 0.801, sensitivity 71.4%, specificity 95.0%, Youden index = 0.601) (Fig. 2B). The ROC analysis excluded “age”, since it represents an independent variable.

**Table 3:** Multivariate linear regression model with GA as dependent variable

	Estimate	Standard Error	CI 95%	*P*-value
**Age**	0.049	0.012	0.025–0.073	< 0.001
**BMI**	0.054	0.037	-0.017- 0.127	NS
**FPG**	0.070	0.005	0.061–0.079	< 0.001
**Basal Insulin**	-0.013	0.027	-0.067-0.041	NS
**Creatinine**	0.843	0.590	-0.320-2.007	NS

*FPG* fasting plasma glucose, *BMI* body mass index, *NS* not statistically significant

## Discussion

Our study lays the foundations to consider GA as a useful marker in diagnosing DM in TDT patients. Indeed, TDT patients may frequently develop DM [6, 30], a condition which necessitates strict clinical and laboratory monitoring to protect patients from the increased risk of micro- and macrovascular complications [31]. Frequent transfusions and alterations in the normal red blood cell turnover reduce the ability of HbA1c to accurately reflect the diabetic condition [32, 33]. To improve the possibility of early DM diagnosis Good Clinical Practice of the Italian Society of Thalassemia and Haemoglobinopathies suggests that in TDT population OGTT should be performed once a year [20], although this is still a matter of debate because may be challenging in clinical practice [34]. Indeed, OGTT presents some limitations: first of all, it can be challenging for patients to follow, as it requires a controlled fasting period and the consumption of a glucose solution, which may cause discomfort or nausea. Furthermore, OGTT may not always detect impaired glucose metabolism in its earliest stages, potentially delaying diagnosis and intervention [35]. Finally, in the TDT population, DM exhibits a unique pattern resulting from a combination of insulin deficiency and insulin resistance. Consequently, during an OGTT, blood glucose levels may appear normal at 120 min, potentially masking an underlying impairment in insulin secretion [12]. However, this dysfunction can become evident earlier in the test: an elevated glucose level at 60 min or a slight increase in FPG indicates a delayed and abnormal insulin response [10]. For a comprehensive assessment of the OGTT response and to define the potential role of GA in TDT patients, in addition to classical parameters, we also evaluated glucose levels at 60 min under OGTT and AUC_G_. These parameters indeed correlated with GA levels, suggesting that the latter may be a useful marker for DM diagnosis.

Previous studies proposed fructosamine as a marker for assessing DM in TDT. However, in the general population fructosamine levels did not significantly differ between individuals with normal glucose tolerance (NGT) and those with impaired fasting glucose (IFG) or DM [36]. Along this line, we found that fructosamine was not useful in assessing glycaemic status, since it failed to discriminate NGT from DM patients in our TDT cohort. This finding may be attributed to the considerable variability in fructosamine levels, which are influenced by inflammation, infections and chronic diseases [37]. Therefore, fructosamine is unlikely to be useful in TDT patients where the latter conditions are very frequent.

Recently, the use of GA has been proposed for DM monitoring. Unlike HbA1c, it is not influenced by frequent transfusions and provides a picture of average blood glucose levels over the previous 2–3 weeks. These characteristics make GA a potentially reliable marker in thalassaemic patients [37, 38]. Additionally, GA has proven to be a more sensitive timely biomarker than HbA1c in reflecting postprandial hyperglycaemia [15, 39, 40].

Our study evaluates for the first time the role of GA in the diagnosis of DM in TDT patients. We indeed found a significant correlation between GA levels and both fasting and post-OGTT glucose concentrations. The ROC analyses indicated a GA cut-off providing excellent sensitivity and specificity in identifying DM patients, supporting the hypothesis that GA may be a reliable marker for screening glucose metabolism disorders in TDT population. The lower sensitivity of GA cut-off identified by using OGTT vs. FPG data may be due to the altered insulin response and sensitivity in TDT patients. Additionally, previous studies in β-T suggested that iron deposition and inflammation within the pancreatic islets may damage both α and β cells, disrupting the release of both insulin and glucagon [41]. This, in turn, complicates the understanding of DM pathogenesis in this cohort, highlighting the challenges in standardizing a diagnostic tool that accurately reflects the metabolic responses in individuals with TDT [42].

In our cohort, GA levels were not influenced by sex, consistent with the findings obtained from several studies conducted in the general population [40, 43]. On the other hand, GA levels appear to increase significantly with age. In the TDT population, this trend could be partially attributed to chronic iron exposure [44]. As a matter of fact, in a genetically determined condition such as β-T, reaching an advanced age implies a longer history of transfusions and, consequently, a greater cumulative iron exposure [45]. However, since a similar pattern is observed in the general population, it is also likely to be linked to physiological changes associated with aging [19]. These factors collectively facilitate non-enzymatic glycation processes, contributing to elevated GA levels over time [46]. Furthermore, Pre-transfusion Hb levels did not showed significant association with GA in univariate and multivariate models. This suggests that GA diagnostic value is independent of transfusion regimen variations within our cohort. It is worth noting that all patients were managed according to standardized transfusion protocols aimed at maintaining pre-transfusion Hb levels between 9.5 and 10.5 g/dL, in line with international recommendations. The low variability in Hb levels hampers the detection of the effects of Hb levels fluctuations/transfusion burden on GA values.

In our study, we observed a weak positive correlation between GA levels and BMI. This could be related to increased insulin resistance associated with adipose tissue accumulation, as well as chronic inflammation linked to obesity, which plays a crucial role in promoting non-enzymatic glycation processes [47]. This suggests that in overweight or obese patients, higher GA levels may reflect not only impaired glucose metabolism but also the broader metabolic disturbances characteristic of obesity, further emphasizing the need to account for BMI when interpreting GA levels in clinical practice [39, 48]. Conversely, Koga et al. [47] found a negative correlation between BMI and serum levels of both albumin and GA, suggesting that chronic inflammation associated with obesity accelerates albumin turnover process, increasing albumin degradation and reducing the production of advanced glycation end-products [49, 50]. In addition, Huh et al. [51] found that in the general population BMI negatively correlates with GA in NGT subjects but not in DM patients, potentially underestimating glucose metabolism control. However, these findings were referred to the general population and not to TDT patients, where obesity is an uncommon condition. As a result, while BMI-related metabolic disturbances may be relevant in other populations, their role in GA regulation is likely less important in TDT subjects.

We found that GA positively correlates with basal insulin and HOMA-IR index, suggesting that GA may mirror insulin sensitivity in TDT populations. On the other hand, GA does not correlate with HOMA-β, suggesting that GA does not provide information on β-cell function and could not be useful to detect insulin deficiency [52]. This finding is in line with recent evidence showing that in the general population HOMA-β does not correlate with GA levels [45]. All together, these findings are in line with the lack of correlation between GA and pancreatic iron overload. The absence of this correlation can be explained by several mechanisms related to pancreatic iron compartmentalization and compensatory responses [53]. First of all pancreatic iron accumulation primarily affects β-cells, which represent less than 2% of the total pancreatic mass. This focal damage may not significantly impact overall insulin secretion until advanced stages of iron overload are reached. Furthermore, early pancreatic iron deposition may trigger compensatory hyperinsulinemia from unaffected β-cells, potentially masking glycemic deterioration that would otherwise be reflected by GA levels [54]. The temporal dissociation between pancreatic iron accumulation and functional impairment adds another layer of complexity to this relationship. GA reflects 2–3 weeks of glycemic control, while pancreatic T2* represents cumulative iron burden accumulated over years. Early iron accumulation may precede functional impairment detectable by GA, creating a lag period where structural damage is present but metabolic dysfunction has not yet manifested [55]. Finally, a delay in pancreas iron overload mobilization in patients under chelation therapy compared to liver and heart was found [53].

On the other hand, patients with LIC indicative of moderate to severe liver iron load presented higher GA levels. This correlation underscores the impact of hepatic iron overload on glucose metabolism. Hepatic iron accumulation impairs hepatic insulin clearance, leading to systemic insulin resistance, which contribute to elevated glucose levels reflected in GA [47]. Additionally, hepatic iron overload may affect albumin synthesis and turnover, potentially influencing GA formation kinetics and explaining the observed correlation between liver iron content and GA levels [2]. Indeed, elevated insulin levels and insulin resistance prior to DM onset may be due to a decline in hepatic insulin clearance [48]. Over time, insulin resistance caused by hepatic iron overload can contribute to pancreatic damage [49].

Furthermore, patients with significant heart iron accumulation exhibited markedly higher GA levels. Cardiac muscle represents one of the largest insulin-sensitive tissue masses in the body, and iron-induced oxidative stress in cardiac myocytes may reflect broader skeletal muscle insulin resistance affecting glucose metabolism [56]. In TDT patients iron accumulation in muscle tissue plays an underestimated role in the development of insulin resistance. Previous studies have shown that iron deposition in the skeletal muscle promotes the generation of reactive oxygen species, which disrupt the normal intracellular insulin signalling pathways, thereby reducing insulin efficacy [57]. Moreover, the damage to mitochondrial structures induced by iron impairs both glucose uptake and its utilization by muscle cells [58]. These alterations likely contribute to insulin resistance and further impair glucose metabolism in TDT patients.

These reports further strengthen the potential role of GA as a marker of glucose metabolism derangements in TDT patients, where HbA1c cannot be considered as a reliable tool.

In addition, we found a positive association between GA levels and serum creatinine levels. Consistent with these findings, several studies in the literature have highlighted the potential role of GA as an independent marker of diabetic nephropathy development [59, 60].

## Conclusion

Our study provides the basis to consider GA as a highly effective and reliable marker for DM diagnosis in TDT population. GA levels > 14.95% have demonstrated strong predictive value for DM diagnosis, reinforcing its role as a diagnostic tool and supporting its use as a valuable alternative to HbA1c. These results suggest that integrating GA into clinical practice could enhance early DM diagnosis in TDT, with significant implications for personalized disease management. Our study provides a strong starting point for future multicentre research aimed at establishing GA as a standard reference for glycaemic assessment in thalassaemic patients. Finally, the potential role of GA as a predictor of nephropathy in DM patients represents an additional avenue for further investigation. A deeper understanding of its implications could help refine strategies for preventing metabolic complications and improving clinical outcomes in this high-risk population. The limited availability of published data on this topic underscores the innovation and significance of our research. 

## Supplementary Information

Below is the link to the electronic supplementary material.

Supplementary Material 1

## Data Availability

No datasets were generated or analysed during the current study.
