# Supplementary material for: Glycated albumin as a diagnostic tool for diabetes mellitus in transfusion-dependent β-thalassemia patients
Source: Endocrine. 2025 Sep 9;90(3):1187–96. doi: 10.1007/s12020-025-04410-9 (PMC12708767; doi:10.1007/s12020-025-04410-9)
Supplement: Supplementary file 1 — Supplementary Material 1 [file 12020_2025_4410_MOESM1_ESM.docx]

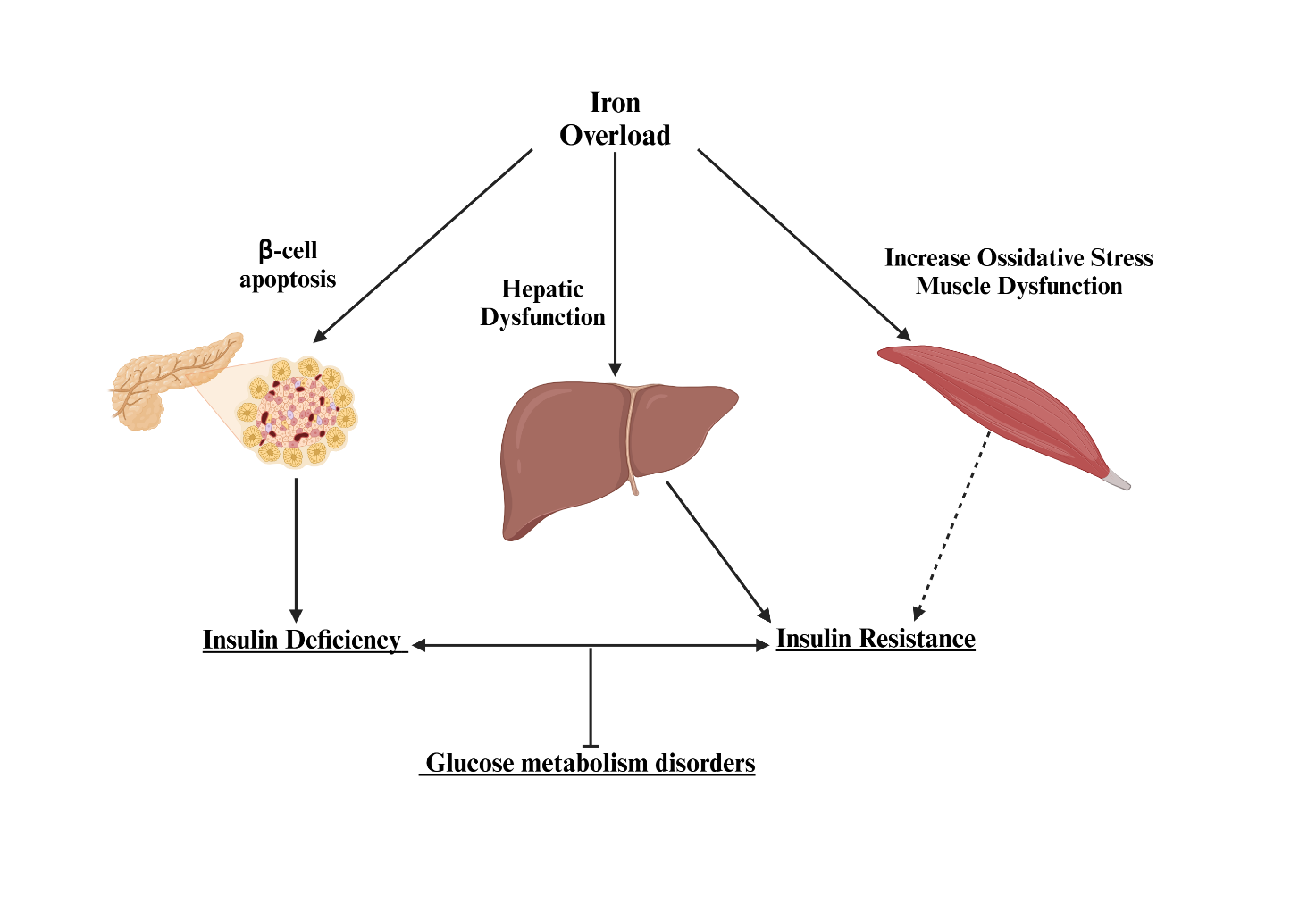


**Figure 1**: The possible pathophysiology of glucose metabolism disorders in TDT.

It illustrates the interplay between key organs involved in glucose homeostasis, highlighting how iron overload disrupts normal glucose homeostasis, potentially leading to conditions such as impaired glucose tolerance (IGT) and DM.

**Figure 2A**: comparing GA levels between individuals with FPG indicative of DM and those with FPG ≤125 mg/dl. **Figure 2B**: comparing GA levels between individuals with G’120 indicative of DM and those with G’120 <200 mg/dl.

Abbreviations: GA= Glycated Albumin FPG= Fasting Plasma Glucose, G’120’= glucose level after 120' from oral glucose administration.

**Figure 3A**: Fructosamine correlation with FPG (r = 0.211, p < 0.01); **Figure 3B**: comparing fructosamine levels between individuals with FPG indicative of DM and those with FPG ≤125 mg/dl.

Abbreviations: FPG= Fasting Plasma Glucose

|  | **Total**  **N=142** | **Female**  **N=74** | **Male**  **N=68** | **p-value** |
| --- | --- | --- | --- | --- |
| **Age (years)** | 50.10±9.09 | 47.10±10.40 | 47.79±9.70 | NS |
| **BMI (kg/m^2^)** | 24.12±3.15 | 23.79±3.20 | 23.38±3.10 | NS |
| **Pre-transfusion Hb (g/dL)** | 10.20±0.76 | 10.20±0.76 | 10.20±0.77 | NS |
| **Transfusion frequency (days)** | 18.00±3.90 | 18.10±3.60 | 17.90±3.10 | NS |
| **Ferritin (ng/ml)** | 749±607 | 780±603 | 750±600 | NS |
| **GA (%)** | 14.20±2.70 | 14.25±3.05 | 13.98±2.45 | NS |
| **OGTT 120’ (mg/dl)** | 154±44.8 | 155±40.8 | 153±49.0 | NS |
| **OGTT 60’ (mg/dl)** | 184±46 | 98.99±26.16 | 100.77±29.55 | NS |
| **Basal Insulin (µU/ml)** | 6.53±4.76 | 6.75±4.40 | 6.69±4.51 | NS |
| **Homa- IR** | 1.69±1.17 | 1.69±1.21 | 1.70±1.19 | NS |
| **HOMA-β (%)** | 90.56±90.56 | 88.75±80.09 | 91.27±80.48 | NS |
| **Total Cholesterol (mg/dl)** | 130.95±33.76 | 130.40±34.30 | 131.33±33.94 | NS |
| **HDL-cholesterol (mg/dl)** | 44.21±11.56 | 45.13±10.89 | 43.55±11.10 | NS |
| **Triglycerides (mg/dl)** | 104.1±58.97 | 106.21±59.25 | 101.11±58.73 | NS |
| **Albumin (g/dl)** | 4.44±0.34 | 4.09±0.32 | 4.76±0.48 | **0.039** |
| **Creatinine (mg/dl)** | 0.75±0.21 | 0.75±0.18 | 0.79±0.19 | NS |
| **eGFR (ml/min)** | 105.29±22.13 | 100.10±26.20 | 99.33±25.22 | NS |

**Table 1. OGTT Patients characteristics.**

Variables are shown as mean ± standard deviation.

Abbreviations: NS= not statistically significant, Hb=Haemoglobin, BMI=Body Mass Index, GA= Glycated Albumin, FPG= Fasting Plasma Glucose, OGTT 60’= glucose level after 60’ from oral glucose administration; OGTT 120’= glucose level after 120' from oral glucose administration. AUC_G_ = Area under glycaemic curve EGFR= Estimated Glomerular Filtration Rate.
